# Adrenaline-induced hypokalemia and critical QT prolongation in a patient with refractory anaphylactic shock complicated by myocardial ischemia: a case report

**DOI:** 10.1097/MS9.0000000000005294

**Published:** 2026-06-30

**Authors:** Chikako Wada, Motohiro Sekino, Ryosuke Shintani, Shohei Kaneko, Akihiro Yokoyama, Yusuke Kasai, Naoya Iwasaki, Hiroshi Araki, Rintaro Yano, Taiga Ichinomiya, Ushio Higashijima, Tetsuya Hara

**Affiliations:** a Department of Anesthesiology and Intensive Care Medicine, Nagasaki University Graduate School of Biomedical Sciences, Nagasaki, Japan; b Department of Intensive Care Medicine, Saga University Hospital, Saga, Japan

**Keywords:** acute coronary syndrome, anaphylaxis, arrhythmia, epinephrine, potassium

## Abstract

**Introduction:**

Adrenaline is the first-line treatment for anaphylactic shock. However, adrenaline administration may exacerbate myocardial ischemia and induce fatal arrhythmias. It can also cause hypokalemia, thereby increasing the risk of life-threatening cardiac events.

**Case presentation:**

A 58-year-old man developed refractory anaphylactic shock accompanied by electrocardiographic evidence of myocardial ischemia during spinal fusion surgery. A total of 3.6 mg of intravenous adrenaline was required. At 135 minutes after onset, the serum potassium level decreased to a nadir of 2.1 mmol/L, resulting in critical QT prolongation. Timely adrenaline discontinuation and potassium supplementation resolved the hypokalemia, resulting in a favorable outcome.

**Discussion:**

Adrenaline is known to induce hypokalemia through a rapid and severe intracellular potassium shift via β2-adrenergic stimulation; however, this effect may be underestimated. The synergistic interaction of myocardial ischemia during anaphylactic shock, the direct adverse effects of adrenaline on the myocardium, and adrenaline-induced hypokalemia significantly increases the risk of fatal arrhythmias.

**Conclusion:**

When using multiple and/or continuous doses of adrenaline, clinicians should consider proactive monitoring of potassium levels and potassium supplementation, thereby preventing life-threatening arrhythmias.

## Introduction

Adrenaline is the first-line treatment for anaphylactic shock owing to its potent α- and β-adrenergic properties and inhibitory effects on the release of mediators[1]; it is also used to treat cases complicated by myocardial ischemia, such as Kounis syndrome^[^2,3^]^. However, its adrenergic effects may exacerbate myocardial ischemia and induce fatal arrhythmias^[^4,5^]^. Moreover, adrenaline induces hypokalemia through β2-adrenergic effects[6]. When adrenaline-induced hypokalemia occurs in patients with anaphylaxis with ischemic and hypersensitive myocardium, it causes QT prolongation and, ultimately, torsades de pointes, ventricular tachycardia, and ventricular fibrillation^[^7,8^]^. While the physiological mechanism of adrenaline-induced hypokalemia is well established[6], its clinical significance during the management of refractory anaphylaxis is insufficiently emphasized in previous case reports and current anaphylactic guidelines^[^1,9–12^]^. We report this case to highlight the critical risk of severe hypokalemia and subsequent QT prolongation when high-dose adrenaline is warranted.

HIGHLIGHTS

- A case of severe adrenaline-induced hypokalemia in a patient with anaphylactic shock and myocardial ischemia is reported.
- High-dose adrenaline resulted in a potassium nadir of 2.1 mmol/L and QT prolongation.
- Adrenaline-induced hypokalemia may increase the risk of fatal arrhythmias, particularly in patients with myocardial ischemia.
- Clinicians should consider frequent potassium monitoring during repeated or continuous adrenaline administration.
- Proactive potassium supplementation may help prevent life-threatening arrhythmias in this clinical setting.

## Case presentation

A 58-year-old man with no history of allergies was scheduled to undergo spinal fusion surgery at an orthopedic hospital, where anesthesia was managed by a part-time anesthesiologist, and point-of-care testing for electrolytes was unavailable. The patient presented with hypertension, treated with calcium channel and angiotensin receptor blockers; preoperative laboratory results, including potassium (3.8 mmol/L), were normal.

**Figure 1.**
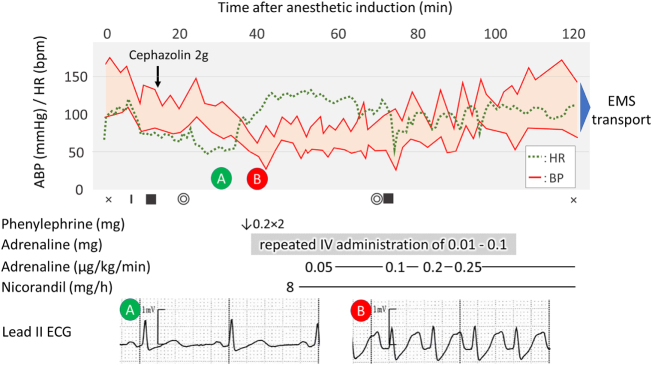
Anesthesia record. Twenty minutes after the initiation of surgery, the patient developed refractory shock. The electrocardiogram waveform, which was normal prior to the onset of shock (A), shows ST-segment depression at the time of shock onset (B). Combined with bronchospasm, this led to a clinical diagnosis of Kounis syndrome. In addition to frequent intravenous injections of adrenaline, a continuous intravenous infusion of 0.25 μg/kg/min was required. During the course of treatment, premature ventricular contractions and sudden bradycardia repeatedly occurred. ×, Anesthesia start and end; I, intubation; ◎, surgery start and end; ■, position change (prone and supine position); BP, blood pressure; bpm, beats per minute; ECG, electrocardiogram; EMS, emergency medical service; HR, heart rate; IV, intravenous.

General anesthesia was induced by administering atropine, thiamylal, remifentanil, and rocuronium, followed by inhalation maintenance with sevoflurane, continuous infusion of remifentanil, and administration of cefazolin. Twenty minutes after surgery initiation, the patient developed shock (Fig. 1). Fluid resuscitation with a balanced crystalloid and phenylephrine was unsuccessful. Simultaneously, bronchospasm and ST-segment depression in lead II were observed on electrocardiography (Fig. 1), leading to a clinical suspicion of Kounis syndrome.

Adrenaline 0.01 mg was administered intravenously several times. Subsequently, 0.05 mg of adrenaline, followed by multiple 0.1 mg doses, was administered, yielding minimal response. Then, a continuous infusion of adrenaline via a peripheral vein at 0.05 μg/kg/min was initiated. Additionally, nicorandil, a coronary vasodilator (8 mg/h), and methylprednisolone (125 mg) were administered. Owing to refractory shock, the surgery was terminated, and the patient was transferred to our hospital. When the patient returned to the supine position, erythema (which was absent at onset) was observed over the entire body surface. Following arterial and central line insertion, the patient was transferred under continuous adrenaline (0.25 μg/kg/min), nicorandil, midazolam, and fentanyl administration. Despite improved ST-segment depression and stable blood pressure, recurrent premature ventricular contractions and bradycardia occurred. A cumulative dose of 3.6 mg of adrenaline was administered over 135 minutes due to the refractory nature of the anaphylactic shock, which did not respond to initial standard bolus doses. Given the limited resources at the primary orthopedic hospital and the need to maintain systemic perfusion during the 135-minute interval until intensive care unit (ICU) admission at our tertiary center, a continuous infusion was maintained as a life-saving measure.

**Figure 2.**
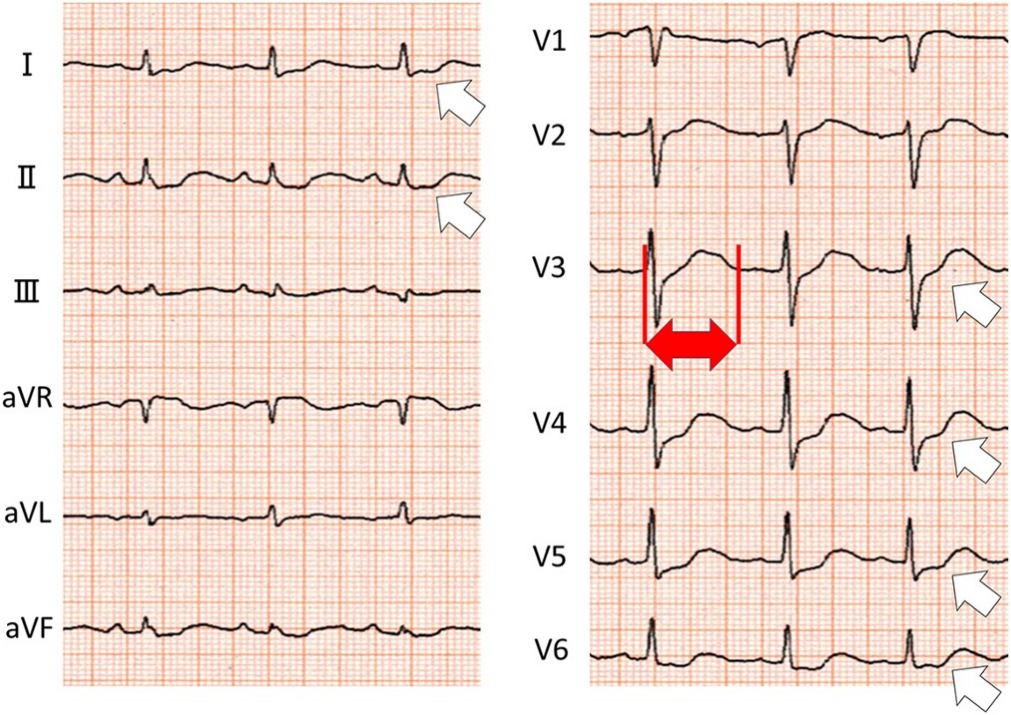
Twelve-lead electrocardiography: Critical QT prolongation (corrected QT time 0.581 s, using Bazett’s formula) (double-headed red arrow in lead V3) and diffuse ST-segment depression (white arrows) were confirmed.

Upon ICU admission, laboratory analyses revealed severe hypokalemia (2.1 mmol/L). A 12-lead electrocardiogram confirmed critical QT prolongation (corrected QT interval of 0.581 s using Bazett’s formula) and diffuse ST-segment depression (Fig. 2) (Table 1). Serum ionized calcium level was slightly decreased at 1.12 mmol/L, whereas the levels of magnesium (1.8 mg/dL) and all other electrolytes were within normal ranges. The patient exhibited lactic acidosis and hyperglycemia (Table 1). Creatine kinase-MB was slightly elevated at 23 U/L; however, echocardiography revealed no abnormalities in cardiac wall motion.

**Table 1:** Laboratory data of the patient before surgery and after ICU admission.

	Before surgery	Time course after ICU admission (135 min after onset)						
		0 h	2 h	4 h	8 h	12 h	24 h	36 h
Potassium, mmol/L	3.8	2.1	2.2	3.2	3.8	4.1	3.8	4.1
QTc interval, s	0.390	0.581	0.580	0.586	0.524	0.453	0.427	0.405
Lactate, mmol/L	–	6.9	8.8	8.1	5.1	4.5	2.6	1.2
Base excess, mmol/L	–	−4.5	−5.0	−3.0	−0.5	−0.4	1.0	0.3
Glucose, mg/dL	87	250	299	221	155	162	148	114
Adrenaline, µg/kg/min	0	0.25	0.15	0.01	0	0	0	0

QTc, corrected QT; ICU, intensive care unit.

Given the severity of hypokalemia and QT prolongation, intravenous potassium chloride (KCl) supplementation was immediately initiated at 8 mEq/h. The target potassium threshold was set at 4.0 mmol/L, and levels were monitored every 1–2 hours. Magnesium sulfate and calcium chloride were also administered, and sedation was maintained with propofol and dexmedetomidine.

After the addition of noradrenaline at 0.2 μg/kg/min and vasopressin at 1.8 U/h, continuous adrenaline infusion was discontinued approximately 4 hours after admission. To avoid rebound hyperkalemia, KCl supplementation was gradually discontinued. Twelve hours after admission, the potassium level reached 4.1 mmol/L, and QT prolongation improved (Table 1). Lactic acidosis and hyperglycemia also normalized. ST-segment depression also improved, and no life-threatening arrhythmias were observed. Based on the clinical presentation (including medical history, symptoms, and treatment response) suggestive of Type I Kounis syndrome characterized by vasospasm, the consulting cardiologist determined that emergency coronary angiography was not indicated. The patient was extubated 22 hours after admission and transferred back to the referring hospital on the 4th day of ICU admission without complications.

Blood test results available later revealed total serum tryptase levels of 43.8 µg/mL at 2 hours and 8.7 µg/mL at 30 hours after onset, confirming the diagnosis of anaphylaxis. However, subsequent skin tests (prick and intradermal) for the agents used prior to the onset, along with serum latex-specific IgE testing, were negative. Consequently, the patient declined further surgical intervention or a detailed coronary workup. No cardiovascular abnormalities have been detected throughout the 1-year outpatient follow-up period.

## Discussion

The management of anaphylactic shock complicated by myocardial ischemia, such as Kounis syndrome, remains challenging. Adrenaline administration may exacerbate myocardial ischemia and induce fatal arrhythmias^[^4,5^].^ Therefore, clinicians should limit adrenaline administration to patients with shock and/or laryngospasm[5], conduct careful hemodynamic monitoring^[^3,4^]^, use the minimum necessary dose[2], and concurrently administer coronary vasodilators^[^2–4^]^. However, to our knowledge, warnings regarding adrenaline-induced hypokalemia that trigger QT prolongation and fatal arrhythmias are lacking in both clinical guidelines and case reports of anaphylaxis. Furthermore, in acute emergency settings, electrolyte assessment may be given lower priority, resulting in potential delays.

Hypokalemia is common in hospitalized patients, typically resulting from renal or gastrointestinal loss[8]. Adrenaline, a potent β2 agonist, induces rapid intracellular potassium shifts via Na^+^/K^+^-ATPase activation[6]. Healthy volunteer studies show that adrenaline infusion >0.08 μg/kg/min causes dose-dependent hypokalemia and QT prolongation[13]. Furthermore, an association between high serum adrenaline levels and hypokalemia has been demonstrated in patients with severe trauma, suggesting that stress-induced endogenous adrenaline secretion may contribute to hypokalemia[14]. Considering other perioperative contributors to hypokalemia[8], we noted that the patient had not received β-agonist bronchodilators, loop diuretics, insulin, or alkalizing agents. Although corticosteroids were administered and could have contributed to the electrolyte imbalance, the rapid onset of hypokalemia following adrenaline administration suggests that adrenaline-induced β2-stimulation was a plausible major contributor. In our case, a total of 3.6 mg of adrenaline was administered over 135 minutes, including continuous infusion at 0.25 μg/kg/min, which likely resulted in severe hypokalemia of 2.1 mmol/L. Similarly, a previously reported case of Kounis syndrome with cardiac arrest required multiple doses of adrenaline, resulting in a decrease in potassium levels from 4.8 to 2.2 mmol/L within 56 minutes[15]. Given that severe hypokalemia (<2.5 mmol/L) is a life-threatening condition that may lead to fatal arrhythmia[16], treatment with adrenaline can cause precipitous hypokalemia within a short time.

No intervention has been reported for the prevention of adrenaline-induced hypokalemia. Therefore, frequent monitoring of potassium levels (e.g., every 0.5–1 h) may be prudent to detect rapid electrolyte shifts, particularly during high-dose or continuous adrenaline administration. In our case, failure to measure potassium levels after onset led to the development of severe hypokalemia. Additionally, proactive KCl supplementation aimed at maintaining a target threshold of ≥4 mmol/L could be considered to mitigate the risk of QT prolongation and fatal arrhythmias[17]. Adrenaline dose-dependently causes type B lactic acidosis and hyperglycemia[18]. Indiscriminate administration of sodium bicarbonate or insulin should be avoided, as it may worsen hypokalemia[8]. However, because the mechanism of adrenaline-induced hypokalemia involves intracellular potassium shifts, caution is warranted regarding rebound hyperkalemia after discontinuing adrenaline administration[19].

Although major guidelines advocate intramuscular adrenaline to reduce adverse effects^[^1,11,12^]^, they offer no specific guidance for the management of refractory shock necessitating high-dose intravenous adrenaline. Similarly, perioperative anaphylaxis guidelines for anesthesiologists^[^9,10^]^, which recommend administering intravenous adrenaline, do not mention any precautions regarding high-dose adrenaline administration. Compared with cases occurring outside the hospital, in-hospital drug-induced anaphylaxis is 9.3 times more likely to be refractory to adrenaline[20]. Such cases often require higher adrenaline doses and are associated with high rates of arrhythmias (11.9%), cardiac arrest (42.9%), and mortality (26.2%)[20]. The association between adrenaline-induced hypokalemia and worsening prognosis has not yet been proven. However, not only anesthesiologists and acute care physicians who may manage patients with refractory anaphylactic shock and associated myocardial ischemia, but also other healthcare providers should be aware of adrenaline-induced hypokalemia and its management. This is because, in the chaotic environment of clinical emergencies, electrolyte management is frequently deprioritized in favor of immediate life-saving interventions, potentially leading to a critical delay in diagnosing and treating life-threatening hypokalemia. Furthermore, this case report highlights the need for future observational or prospective studies examining the incidence and prognostic impact of adrenaline-induced hypokalemia.

This case report has several limitations. First, definitive diagnostic procedures, such as coronary angiography and serial troponin measurements, were not performed. Although the clinical presentation was consistent with Type I Kounis syndrome, the patient prioritized returning to daily life and declined further invasive investigations. Second, the long-term cardiac follow-up was limited to clinical observations. Therefore, our conclusions regarding the interplay between adrenaline and myocardial ischemia should be interpreted with caution.

## Conclusion

Herein, we report a case of severe adrenaline-induced hypokalemia and critical QT prolongation in a patient with refractory anaphylactic shock complicated by myocardial ischemia. Adrenaline-induced hypokalemia heightens the risk of fatal arrhythmias; therefore, clinicians should remain vigilant regarding electrolyte shifts, and proactive potassium monitoring and supplementation should be considered during repeated or continuous adrenaline dosing.

## Data Availability

Data sharing is not applicable to this article, as no datasets were generated or analyzed during the current study.
